# Importance of vaccine action and availability and epidemic severity for delaying the second vaccine dose

**DOI:** 10.1038/s41598-022-11250-4

**Published:** 2022-05-10

**Authors:** Luděk Berec, René Levínský, Jakub Weiner, Martin Šmíd, Roman Neruda, Petra Vidnerová, Gabriela Suchopárová

**Affiliations:** 1 Department of Mathematics, Faculty of Science, Centre for Mathematical Biology, University of South Bohemia, Branišovská 1760, 37005 České Budějovice, Czech Republic; 2 Department of Ecology, Biology Centre, Institute of Entomology, The Czech Academy of Sciences, Branišovská 31, 37005 České Budějovice, Czech Republic; 3 CERGE-EI, Politických vězňů 7, 11121 Prague 1, Czech Republic; 4 Siesta Labs, Konopišťská 16, 10000 Prague 10, Czech Republic; 5 The Czech Academy of Sciences, Institute of Information Theory and Automation, Pod Vodárenskou věží 4, 18200 Prague 8, Czech Republic; 6 The Czech Academy of Sciences, Institute of Computer Science, Pod Vodárenskou věží 2, 18200 Prague 8, Czech Republic

**Keywords:** Diseases, Infectious diseases, Viral infection, Health policy

## Abstract

Following initial optimism regarding potentially rapid vaccination, delays and shortages in vaccine supplies occurred in many countries during spring 2021. Various strategies to counter this gloomy reality and speed up vaccination have been set forth, of which the most popular has been to delay the second vaccine dose for a longer period than originally recommended by the manufacturers. Controversy has surrounded this strategy, and overly simplistic models have been developed to shed light on this issue. Here we use three different epidemic models, all accounting for then actual COVID-19 epidemic in the Czech Republic, including the real vaccination rollout, to explore when delaying the second vaccine dose by another 3 weeks from 21 to 42 days is advantageous. Using COVID-19-related deaths as a quantity to compare various model scenarios, we find that the way of vaccine action at the beginning of the infection course (preventing infection and symptoms appearance), mild epidemic and sufficient vaccine supply rate call for the original inter-dose period of 21 days regardless of vaccine efficacy. On the contrary, for the vaccine action at the end of infection course (preventing severe symptoms and death), severe epidemic and low vaccine supply rate, the 42-day inter-dose period is preferable, at any plausible vaccine efficacy.

## Introduction

Although recent modeling studies cast some doubt on earlier optimism that vaccination may mean an end to the COVID-19 pandemic^1,2^, it undoubtedly provides significant leverage that at least temporarily may bring back a return to close-to-normal life. Even so, while everything sounded promising during the autumn 2020 months, delays and shortages in vaccine supplies in spring 2021 led to complications and spurred thinking on strategies that would speed up protection of the public. Indeed, models have clearly demonstrated that vaccine deployment delays substantially affected the course of the pandemic^3,4^. Eventually, the strategy of delaying the second dose, required for all actually available vaccines except the Ad26.COV2-S one produced by Johnson & Johnson^5^, has often been adopted.

This has also been the case in the Czech Republic (also known as Czechia). As of May 8, 2021, over 3.5 million vaccine doses were applied (about 2.5 million first doses and about 1 million second doses), of which nearly 79% were BNT162b2 (Pfizer/BioNTech) and about 10% were mRNA-1273 (Moderna)^6^. Until April 1, 2021, the second dose was delayed after the first one as recommended by the vaccine producers (21 days for BNT162b2 and 28 days for mRNA-1273^7^). Since then, the Czech government allowed the second dose delay for both vaccines to increase to six weeks, which for some time became the standard vaccination scheme in Czechia^8^.

As with many other issues concerning the COVID-19 pandemic, models have been developed to guide formulation of vaccination strategies. The first set of models of this kind focused on how to distribute a limited number of vaccines within the population; that is, which groups of people to prioritize. With the objective to avert as many deaths as possible, a unanimous answer to this question has been to start with vaccinating the oldest age cohorts and then continue by gradually including younger age cohorts, as a consequence of an accelerating age-dependent infection mortality profile^9,10^. This strategy has been adopted by virtually every country. An obvious exception to this has been a preferential vaccination of the health workers directly interacting with COVID-19 patients.

Given the limited supply of vaccines, the strategy of vaccinating more people by delaying the second dose is at a glance natural. Indeed, the trade-off appears to be between vaccinating a given number of individuals with just one dose, despite reduced efficacy and in the hope that more vaccines will arrive shortly, as opposed to vaccinating half that number and holding one dose back for each administered first dose. Accounting for different protection levels reached after the first and second doses, existing modeling studies suggest that such a “dose-sparing” strategy should indeed be considered, as it would avert more cases of COVID-19 and more deaths due to COVID-19 compared with when an original scheme is used^11,12^.

However, the existing models are too simple to give us robust direction regarding how to proceed. They either do not account for epidemic dynamics^12^ or choose between a one-dose scheme and the recommended two-dose scheme administered to half the people, using for many countries then quite unrealistic values of the effective reproduction number ($R=1.8-2.1$)^11^. In parallel with vaccination, an epidemic continues and many people still get infected and eventually immunized, or die. Moreover, the vaccine supply and distribution are themselves dynamic: in the two-dose scheme, we do not set aside one vaccine per any applied one, because others are coming on a more or less regular basis. None of the studies thus appears to model any current epidemic and any more or less realistic vaccine rollout scenario.

Here we explore whether and when delaying the second vaccine dose from 21 days originally suggested for the BNT162b2 vaccine (Pfizer/BioNTech) by additional 3 weeks to 42 days is advantageous, on the basis of the numbers of COVID-19-related deaths averted by June 30, 2021. In doing this, so as to cover many plausible situations, we consider a variety of potential vaccine efficacies, ways of vaccine action, epidemic severities, and (time-dependent) vaccine supply rates. In addition, to provide robust results, we use three different epidemic models that all independently account for real epidemic in Czechia, including the rise and eventual prevalence of the B.1.1.7 variant of SARS-CoV-2 virus early in 2021, and the actual vaccination rollout strategy. All three models are of the SEIR type, the generally agreed framework to model COVID-19, and all explicitly consider both the asymptomatic and presymptomatic infectious classes. That is, once infected, individuals remain non-infectious for a (latent) period. Then, a proportion of them become infectious yet asymptomatic for the rest of infection, while the others become symptomatic following a short presymptomatic (yet already infectious) period. Symptomatic individuals either recover or die, with different models using various other states of infectiousness within this period. Moreover, all models are structured by age of individuals and type of inter-individual contacts. Each model also has a number of unique assumptions and characteristics (Methods).

## Methods

Here we give a short overview of the models we used to address our questions, as well as describe the vaccination rollout scenarios we considered.

### Vaccination scenarios

Two vaccination rollout scenarios are considered, corresponding to delaying the second vaccine dose by either 21 or 42 days. Each scenario provides the daily amount of available first doses (to be) administered to two population groups: general population and health (and other critical infrastructure) workers. Since our models are age-structured (see below), we follow the widely accepted prioritization strategy and in the former group vaccinate according to age, starting with the eldest individuals. It is important to say that the way epidemic further unrolls depends on its severity and the way the vaccine acts, so the temporal dynamics of people vaccinated in each age group, including the times at which younger age classes are allowed, will be scenario-specific (individuals that become infected will not be vaccinated).

The vaccination scenarios have been generated by a vaccination calculator, developed for a practical use by vaccination coordinators in the Czech Republic regions^13^. This calculator plans day-to-day vaccination for each of the assessed groups. Also, it calculates the maximum amount of applied doses by an inter-temporal choice algorithm together with several boundaries. The boundaries were calibrated by empirical data on the actual age-group vaccination in Czechia between December 27, 2020 (start of vaccination) and March 15, 2021, with the scenarios being generated up to July 4, 2021. All data up to March 15, 2021 are therefore matching the reality. The maximal amount of daily administered doses has been set to 1/12 of the available doses at the respective day to account for empirically observed limits of the system. The size of group of (health care) workers was set to 550,000, the rest of the population is vaccinated starting from the oldest ones. Each of the two groups were given $50\%$ of each day’s capacity. For the people who were on a waiting list for the second dose by the date of data generation (March 15, 2021) was the second dose date shifted according to the currently evaluated delay. The amounts of doses, delivered to particular dates, were set according to expected delivery as communicated within government’s strategical documents and the media. See the calculator excel file attached to the reference^13^ for a list of all sources on dose quantity.

### Ways of vaccine action and their timing

Given diverse and steadily appearing reports on how the vaccine may act and to what degree of efficacy, e.g., from the UK^14,15^ or Israel^16^, we consider several possibilities: (1) Effect on infection: vaccinated individuals have reduced chance of infection transmission upon contact with an infectious person, such that the probability to get infected upon such a contact is reduced by the factor $1-v_e$, where $v_e$ is vaccine efficacy. Infected vaccinated individuals have the same further infection course as the non-vaccinated ones, and those that are not infected by an actual contact may get so upon further such contact. (2) Symptoms appearance: vaccine does not affect chance of infection transmission, but when a vaccinated individual gets infected, probability of getting symptoms is reduced by the factor $1-v_e$. (3) Hospital admission: when a vaccinated individual gets infected and shows symptoms, probability of becoming hospitalized is reduced by the factor $1-v_e$. (4) Need of ICU: when a vaccinated individual becomes hospitalized, probability of needing an ICU is reduced by the factor $1-v_e$. (5) Dying of COVID-19: when on the ICU, the probability of dying is reduced by the factor $1-v_e$.

### Model H

One of the models we consider is a deterministic compartmental model the major focus of which is on dynamics of hospitalizations. Because of that, symptomatic individuals may have either mild symptoms and be isolated at home, or have severe symptoms and get hospitalized. Hospitalized individuals are first put on a common bed, with some of them later getting worse and moving to an ICU, where after some time a proportion of individuals die. Individuals that do not go to the ICU recover, and those that improve on the ICU return to a common bed for a while to eventually recover and leave hospital. This model discerns four age cohorts: 0–19 years (children), 20–64 years (adults), 65–79 years (seniors), and 80+ years (elderly).

Vaccination is implemented as follows. Only susceptible individuals are vaccinated, and three sequential vaccination classes are considered. Individuals just getting the first vaccine dose go to the first of these classes that corresponds to no vaccine effect for the first 2 weeks after the first dose. If not infected during this period, they pass to the second class where they stay for until one week after the second dose is administered; vaccine efficacy $v_e^1$ is associated with this class. Finally, if still not infected, they eventually pass to the third class for which vaccine efficacy is $v_e^2 \ge v_e^1$. Individuals that are infected in any vaccine class stay in that class and go through the analogous sequence of events as the infected non-vaccinated individuals. For some simulations, we also assume that vaccine efficacy against infection and symptoms appearance may decline with time: step declines in efficacy by $v_1^w$ and $v_2^w$ are assumed to occur 28 days after the first dose and 90 days after the second dose, respectively. This means that the vaccine efficacy decline after the first dose is relevant only when the 42 days delay in administering the second dose is considered. In this case, five instead of three sequential vaccination classes are considered.

**Figure 1:**
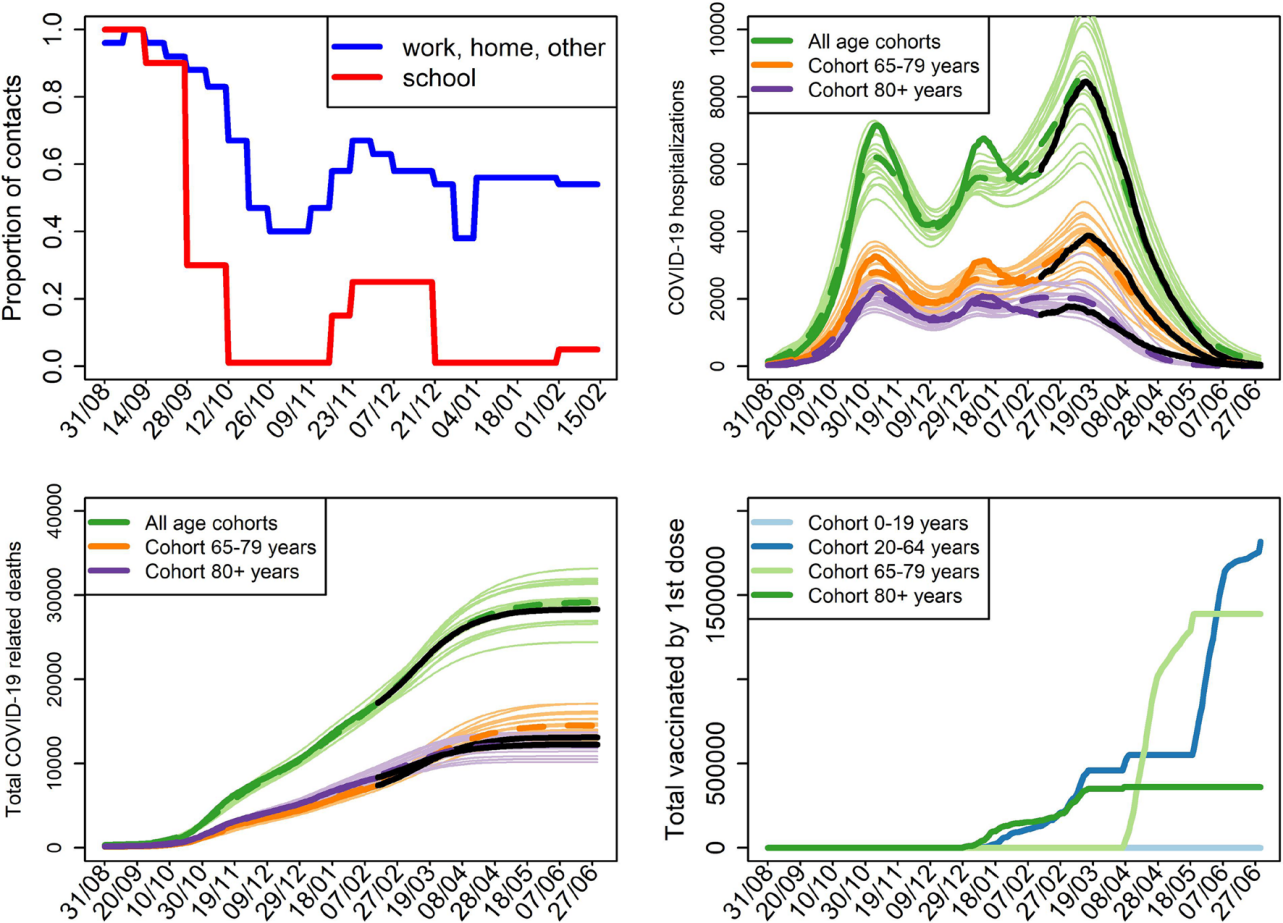
Model H calibration results performed via the Approximate Bayesian Computation technique. The top left panel: proportional reduction of contacts due to COVID-19-related interventions in Czechia; since March 1, 2021, contacts are set at 45% outside schools and 1% in schools. The top right and bottom left panels: while thin lines correspond to the best 20 worlds out of 100.000 simulated ones (selected on the basis of the smallest Euclidean distance between the simulated and observed data), thick dashed lines are respective mean model trajectories. While the thick solid lines are real data up to February 15, 2021, that is, data used for calibration, the black lines are data after that date, providing sound model validation. The bottom right panel: daily numbers of the first vaccine doses administered among the considered age cohorts with 21-day delay.

This model is calibrated on real time series of the actual number of hospitalized individuals and the total number of COVID-19-related death, using the stochastic Approximate Bayesian Computation (ABC) technique^17,18^. More details on this model, including equations, model parameters and specific values used to run it are provided in the Electronic Supplementary Material. In particular, the calibration period is from August 31, 2020, until February 15, 2021. Within this period, epidemic dynamics was largely modulated by temporal changes in the number of social contacts, surveyed throughout all waves of COVID-19 in Czechia^19^; Fig. 1. Then, until March 1, 2021, contacts were set at 55%, respecting results of further rounds of that panel survey. Since March 1, 2021, further restrictions were imposed in Czechia, with sociological data suggesting contacts at 45%. We keep this value until June 30, 2021. We also account for the B.1.1.7 variant of SARS-CoV-2 virus in this model, where we implement its effect as a linear increase in infection transmission by 50% from January 1, 2021, to March 1, 2021. Last but not least, for validation purposes, we assume three weeks difference between the first and second vaccine doses, with $v_e^1 = 0.7$ and $v_e^2 = 0.85$ and the action on infection transmission. A sound fit has been achieved for the model, at the level of both total and age-specific populations (Fig. 1).

### Model M

An alternative model used in the experiments is an agent-based model with agents representing a synthetic population of 56,000 people connected by a realistic network of social contacts. This network comprises 2.7 million edges in 30 layers corresponding to various types of contact, from families and neighborhood to work, school and public transportation. The population and its contact network represent the Hodonin county in the Czech Republic. The underlying epidemic model that runs for every agent is a SEIR-type model with asymptomatic and presymptomatic infectious classes, and a parallel set of states for individuals detected through testing and contact tracing. The model allows for individually assigned parameters based on age, sex, level of protection and other characteristics.

The vaccination is here implemented as a special policy with several parameters operating on the individual level. Non-detected individuals are vaccinated in a stochastic manner according to the given scenario which reflects (age-based) vaccination constrains and preferences. The vaccinated node is marked as vaccinated and has a new counter: the number of days since first vaccine dose. As soon as this counter reaches 14 days, the vaccine efficacy $v_e^1$ is applied. And, as the counter reaches $\delta$ + 7 days (where $\delta$ is the delay between the first and second vaccine shot), actions are implemented with vaccine efficacy $v_e^2$.

**Figure 2:**
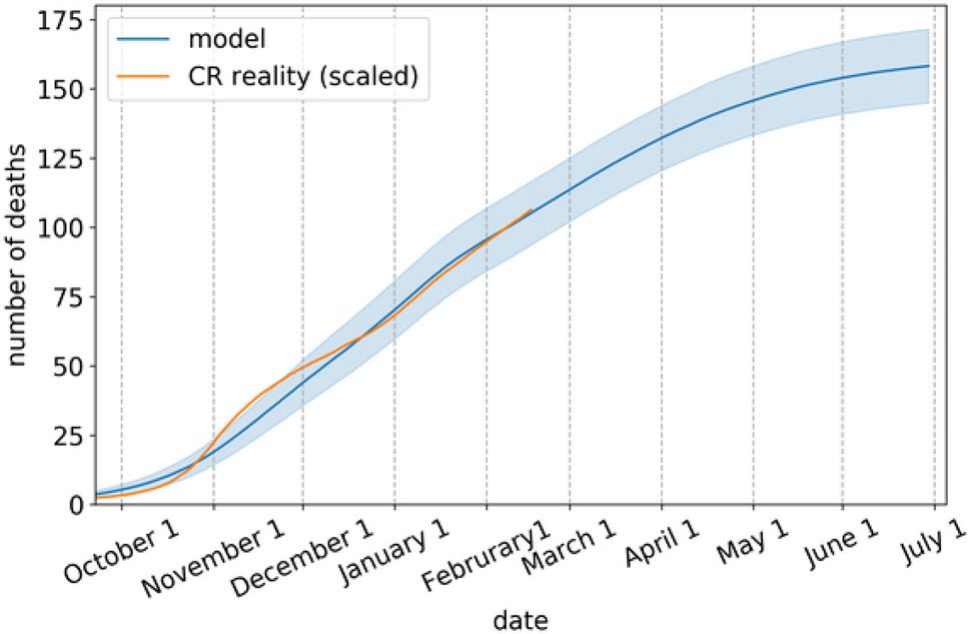
Calibration of Model M. The *y*-axis shows the model-predicted cumulative number of COVID-19-related deaths in simulation (blue, mean ± one standard deviation) compared to the real situation in the Czech Republic scaled to the model population size (orange).

This model is fitted to real time series on COVID-19-related deaths. The calibration was done using extensive grid search. More details about the underlying model and its parameters can be found in^20^. In particular, the model was calibrated on data from October 5, 2021, until February 17, 2021 (Fig. 2). The contact rates in 30 layers were set according to contact reduction in the Czech public given in^19^ and were kept constant from January 24, 2021.

### Model F

Our third model is a stochastic, discrete-time SEIR model distinguishing the same four age cohorts as Model H. This model takes into account both the visible part of the epidemic (i.e. cases revealed by testing) and its hidden part (a certain proportion of asymptomatic and symptomatic cases remain undetected). The course of epidemic is modeled as dependent on the social contact restrictions and the level of fear from epidemic, both surveyed by^19^; thus, the simulated vaccination scenarios are realistic in the sense that they use true values of these determinants.

The vaccination is implemented as follows. First, the probability $\pi _t^i$ of being protected within the *i*-th age cohort at time *t* is determined as $\pi ^i_t = \mu _t^i v^1_e + \nu _t^i v^2_e$, where $\mu _t^i$ and $\nu _t^i$ are the ratios of individuals two weeks after the first dose and one week after the second dose, respectively, and $v^1_e$ and $v^2_e$ are the corresponding efficacies. For the vaccne protection against infection, the force of infection (transition rate from *S* to *E* classes) is simply multiplied by $1-\pi ^i_t$. For the vaccine protection against symptoms appearance variant, the infection rate is left intact yet the probability that a vaccinated person becomes symptomatic after getting infected is multiplied by $1-\pi ^i_t$.

**Figure 3:**
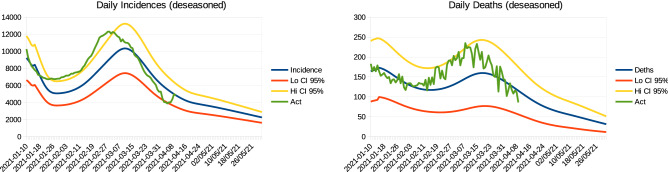
Model F calibration. Left panel: daily detected incidences of COVID-19. Right panel: daily deaths due to COVID-19. Legend: blue line is the mean prediction, while the yellow and red lines delimit the 95% confidence interval. Green curve represents real data.

The model is calibrated by real time series on reported cases, admissions and releases from hospitals, deaths due to COVID-19, and the overall rate of asymptomatic cases revealed by testing and contact tracing. For details, see^21^. In particular, data for calibration cover the period up to April 10, 2021, after which we assumed contact reduction at $50\%$. For the calibration, we assumed (and estimated) different infection transmission and hospitalization rates of the B.1.1.7 variant of the SARS-CoV-2 virus, gradually prevailing since January until March in the Czech Republic. Using vaccine efficacies $v_e^1=0.7$ and $v_e^2=0.9$ of the first and second doses, respectively, calibration and validation results are provided in Fig. 3.

## Results

In Czechia, as in many other countries, the BNT162b2 (Pfizer/BioNTech) vaccine is the dominant one. We thus motivate timing of individual vaccine doses by this vaccine type. The first dose efficacy is commonly reported to establish about 2 weeks after its application, while the second dose efficacy is fully unrolled about one week after its application^16,22^. Since the time intervals between the two doses as well as between the second dose and our time horizon (June 30, 2021) are relatively short, we assume in most of our scenarios that the vaccine efficacy does not wane within these time intervals^2,11^. However, to reflect recent research on vaccine effectiveness waning^23,24^, we alternatively assume that vaccine efficacy against infection and symptoms appearance may indeed decline with time: in such scenarios, step declines by values $v_1^w$ and $v_2^w$ are assumed to occur 28 days after the first dose and 90 days after the second dose, respectively. This means that the vaccine efficacy decline after the first dose is relevant only when the 42 days delay in administering the second dose is considered.

Since our major question is whether and when delaying the second vaccine dose beyond the period recommended by producers (from 21 to 42 days after the first dose) is advantageous, we present our results in a uniform format: given a vaccine action (or a combination of actions), vaccine supply rate and epidemic severity, then for any plausible vaccine efficacy combination we, as our main summary statistic, calculate the amount of extra COVID-19-related deaths among adults of age 65 years or older by June 30, 2021, when adopting the longer delay of 42 days. Negative numbers of extra deaths thus point to the preference of 42 days delay (colored as red in the following figures), whereas positive numbers suggest the 21 days delay preference (colored as blue in the following figures). To account for inevitable uncertainty in model projections, each model is run repeatedly (Models M and F) or over different plausible parameter sets (Model H; Methods) and the average summary statistic is provided.

### Effects of vaccine action

**Figure 4:**
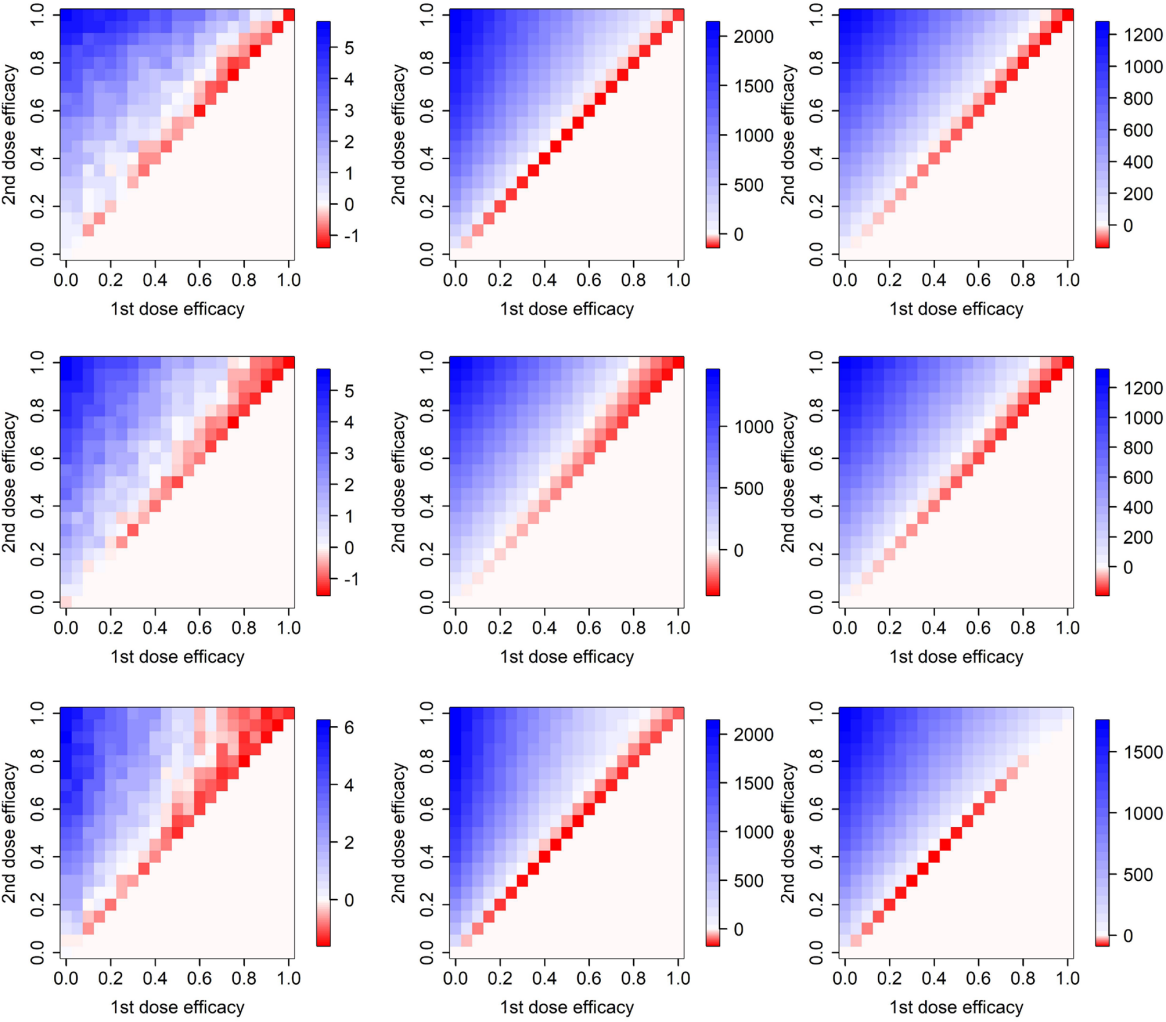
Efficacy of delaying the second vaccine dose by 3 weeks from 21 to 42 days, for different ways of vaccine action and different epidemiological models. Top row: vaccine effect on the probability of getting infected, second row: vaccine effect on the probability of becoming symptomatic when infected, bottom row: combination of effects on infection and symptoms appearance. Left column: model M, middle column: model H, right column: model F.

**Figure 5:**
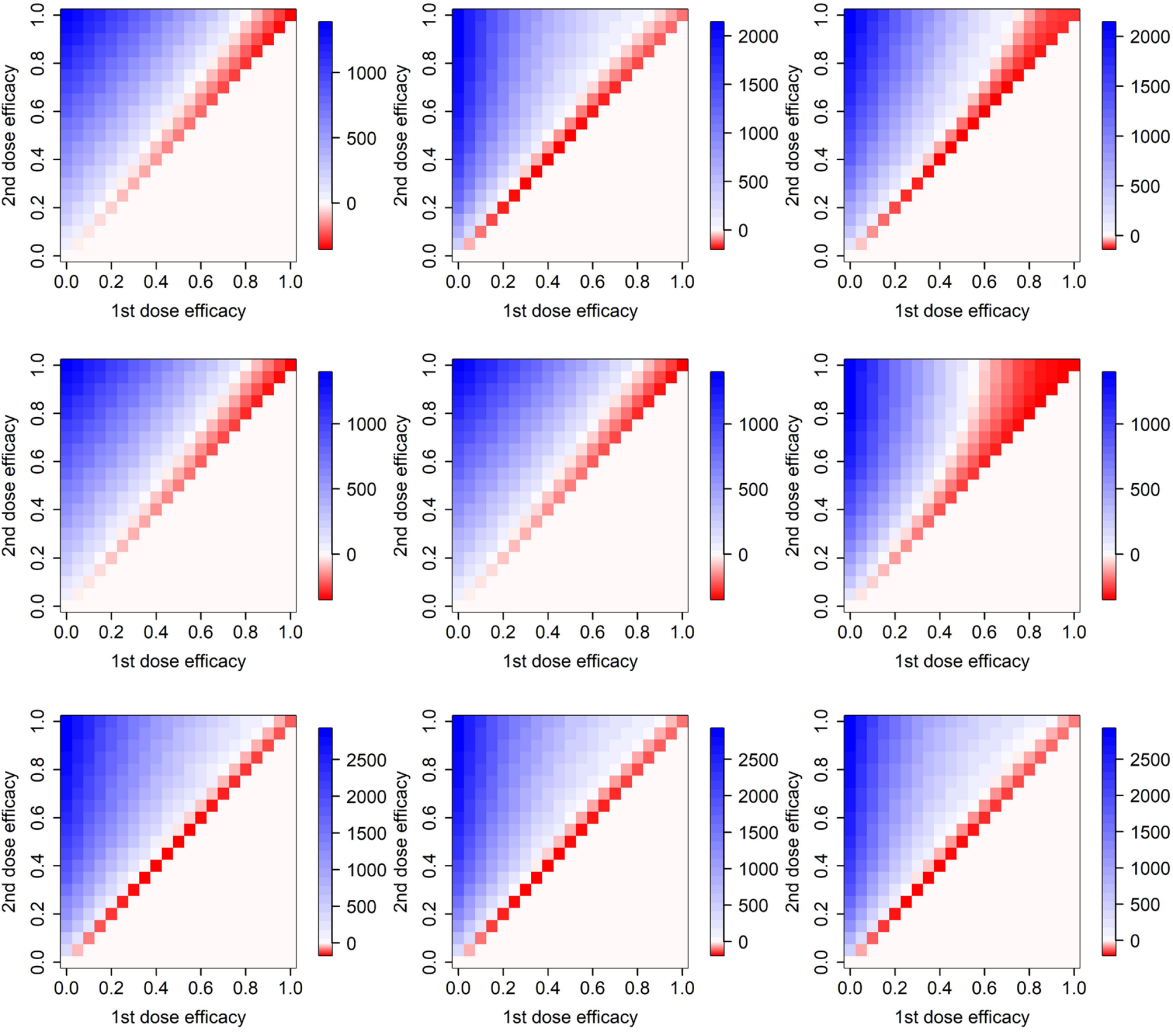
Efficacy of delaying the second vaccine dose by 3 weeks from 21 to 42 days for a variety of vaccine actions. Top row: vaccine effect on the probability that a symptomatic individual becomes hospitalized (left), combination of effects on infection, symptoms appearance and hospitalization (middle), and combination of effects on infection, symptoms appearance, hospitalization, need of ICU and death when on ICU (right). Middle row: vaccine effect on the probability that a hospitalized individual needs ICU (left), dies when on the ICU (middle), and both needs ICU and dies when on ICU (right). Bottom row: vaccine effect on infection and probability of dying (left), on infection, need of ICU and probability of dying (middle), and on infection, hospitalization, need of ICU and probability of dying (right). Only model H is used here.

We consider a number of plausible vaccine actions: protection against infection, symptoms appearance, hospital admission, need of ICU, and death (see Methods for their detailed description). Table 1 summarizes all the combinations we discuss in what follows. Vaccine action has a non-negligible effect on whether it is advantageous to delay the second dose by 42 rather than 21 days (Figs. 4 and 5). Delaying the second dose by 42 rather than 21 days appears most beneficial (largest red area) when the vaccine acts simultaneously on the probabilities of needing an ICU when in hospital and dying when in the ICU (Fig. 5 middle right). On the contrary, the effect of larger vaccine delay seems virtually negligible (largest blue area) when the vaccine acts simultaneously on the earliest two elements in the infection progression (probabilities of transmission and symptoms appearance; Fig. 4 bottom row).

**Table 1:** Combinations of vaccine actions examined in this study.

I	S	H	U	D	Figure
x	-	-	-	-	4, top row
-	x	-	-	-	4, middle row
x	x	-	-	-	4, bottom row
-	-	x	-	-	5, top left
-	-	-	x	-	5, middle left
-	-	-	-	x	5, middle middle
-	-	-	x	x	5, middle right
x	x	x	-	-	5, top middle
x	-	-	-	x	5, bottom left
x	-	-	x	x	5, bottom middle
x	-	x	x	x	5, bottom right
x	x	x	x	x	5, top right

In columns, various vaccine actions are indicated: protection against infection (I), symptoms appearance (S), hospital admission (H), need of ICU (U), and death (D).

Importantly, effects of vaccine action in different steps of infection progression do not add up but rather multiply. This of course follows trivially from the model formulation, but may be surprising at first glance. Just compare the vaccine effect on preventing infection (Fig. 4 top row), preventing symptoms (Fig. 4 middle row) and their combined effect (Fig. 4 bottom row). Or alternatively the effect on preventing symptoms and death (Fig. 5 bottom left), probability of needing ICU (Fig. 5 middle left), and on all these three actions together (Fig. 5 bottom middle). Multiplicity of the ways of action means that when the infection in a proportion of individuals in a model state is averted due to a vaccine action, only a proportion of the infections in the remaining proportion are averted in a following state of infection progression (symptoms appearance, probability of getting hospitalized, needing an ICU, or dying) .

### Effects of epidemic severity

**Figure 6:**
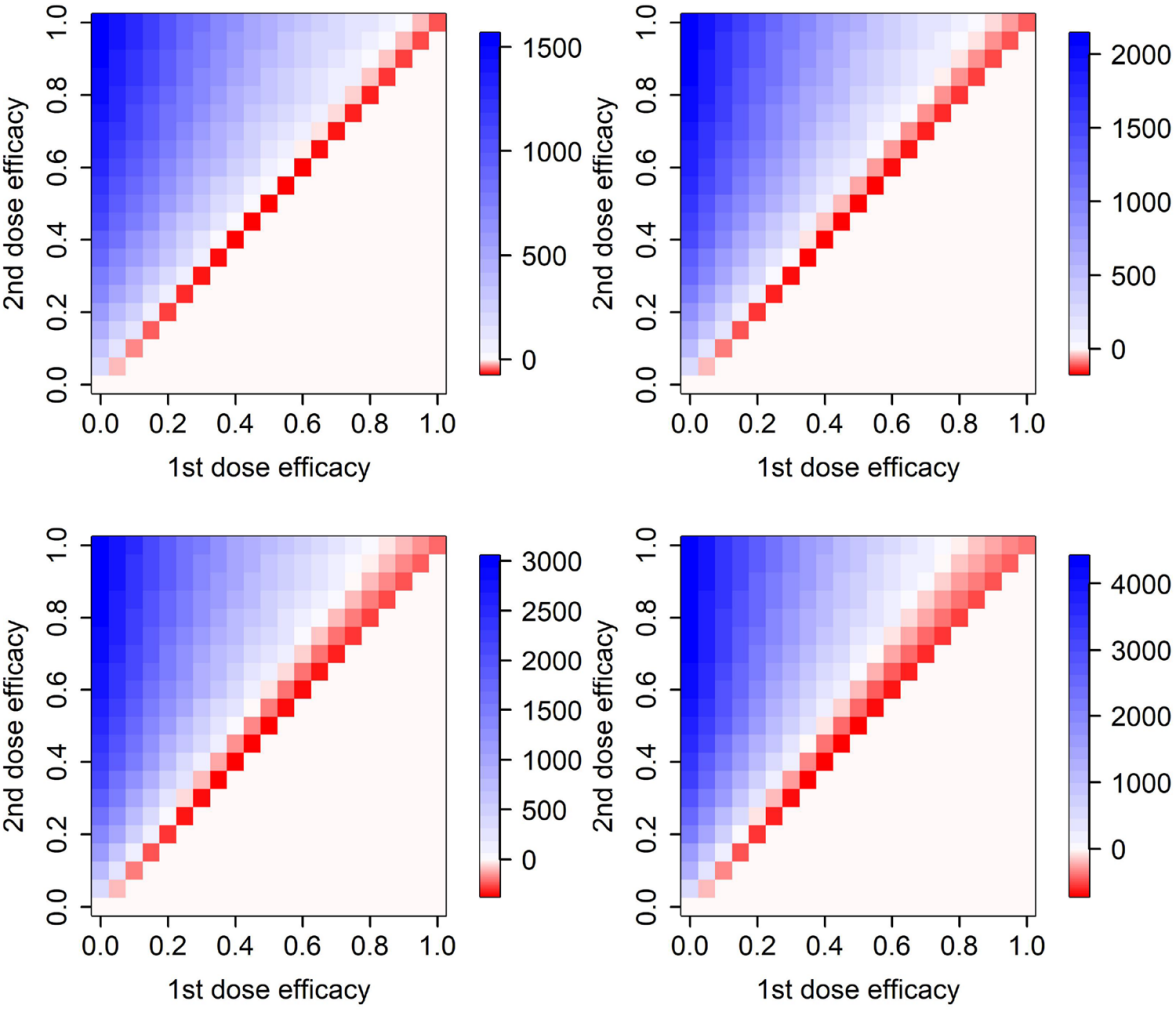
Efficacy of delaying the second vaccine dose by 3 weeks from 21 to 42 days, assuming vaccine effect on the probability that a vaccinated person gets infected by contact with an infectious one, and at the same time on the probability that a vaccinated person becomes symptomatic when infected. Top left: contacts at 35% of the pre-pandemic state, top right: contacts at 45% (real situation in Czechia), bottom left: contacts at 55%, bottom right: contacts at 65%. Only model H is used here.

The above results are based on the actual infection severity of epidemic in the Czech Republic. This is to a large extent driven by the contact structure, estimated to be at 45% of the pre-pandemic state since March 1, 2021 (Methods). An increase in the number of contacts, that is, in epidemic severity, results in a larger set of vaccine efficacy combinations for which it is advantageous to delay the second dose for 42 days (Fig. 6). Importantly, this effect is analogous for any vaccine action (or their combination; just the combination of effects on infection transmission and symptoms appearance is shown here). In conclusion, the more severe the epidemic is, the more advantageous it is to delay the second vaccine dose by 42 days.

We note that an increase in the number of contacts causes an increase in the effective reproduction number. Moreover, since the contact rate and the transmission probability per contact affect the effective reproduction number together as a product, our results stay unchanged if the contact rate is kept at 45% of the pre-pandemic state while the transmission probability per contact varies (e.g., due to more effective personal protection or a novel virus variant).

### Effects of vaccine availability

**Figure 7:**
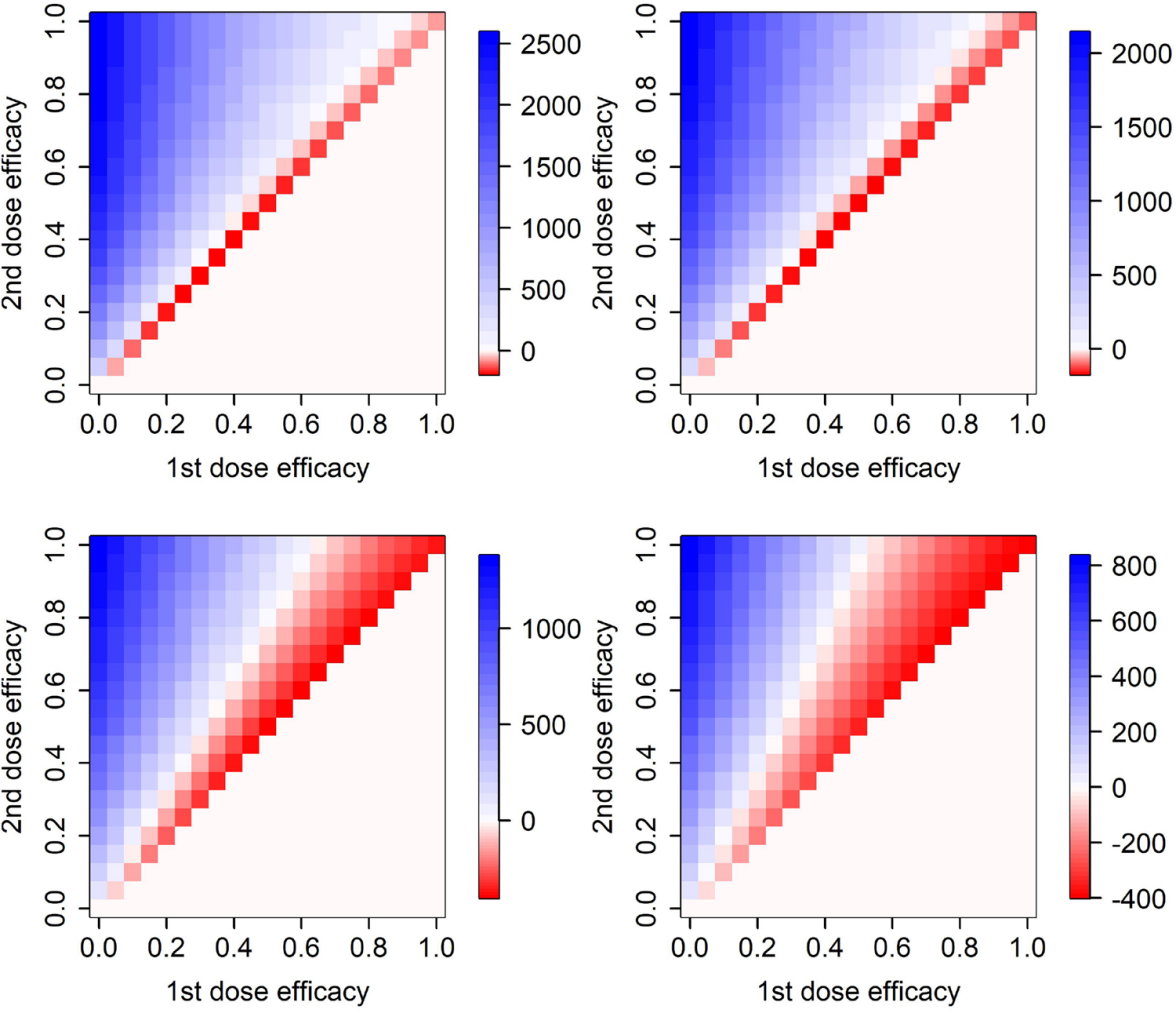
Efficacy of delaying the second vaccine dose by 3 weeks from 21 to 42 days, assuming vaccine effect on the probability that a vaccinated person gets infected by contact with an infectious one, and at the same time on the probability that a vaccinated person becomes symptomatic when infected. Top left: twice more doses available, top right: actual state in Czechia, bottom left: twice less doses available, bottom right: four time less doses available. Only model H is used here.

We also test effects of vaccine supply scenarios that differ from the actual state in Czechia. As Fig. 7 clearly demonstrates, the less vaccines are available the more advantageous it is to delay the second vaccine dose for 42 days. Again, these results do not change qualitatively if other ways of action (or their combinations) are considered. Interestingly, further improvement in the vaccine supply rate relative to the actual vaccination rollout in Czechia does not suggest any significant change in the inter-dose delay preferences, but shortage of supplies, on the other hand, results in much wider vaccine efficacy combinations at which the 42-days delay is advantageous (Fig. 7).

### The extreme scenarios

**Figure 8:**
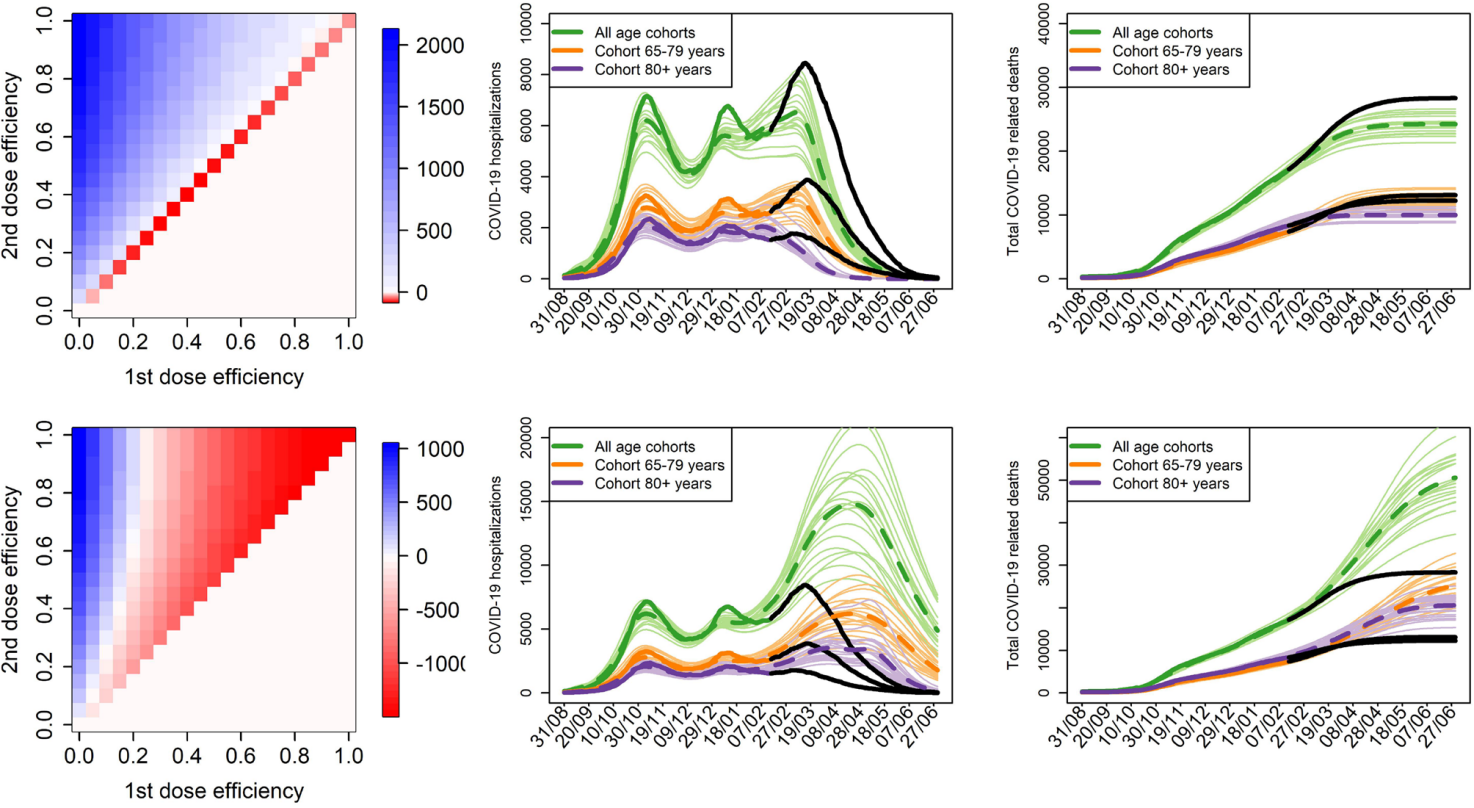
The extreme scenario. Efficacy of delaying the second vaccine dose by 3 weeks from 21 to 42 days, assuming (top row) vaccine effect on infection and the probability that an infected person shows symptoms, contacts at 35% of pre-pandemic state and twice more doses available, and (bottom row) vaccine effect on the probabilities of needing an ICU and dying when on the ICU, contacts at 65% and four times less doses available. Each of these extreme scenarios is supplemented by the course of numbers of hospitalized and dead individuals (legend as in Fig. 1 in Methods). Only model H is used here, with 70% and 90% efficacy of the first and second dose, respectively.

Vaccine effect on infection and probability of having symptoms when infected, mild epidemic and sufficient vaccine supply rate call for the original inter-dose period of 21 days regardless of the vaccine efficacy combination (Fig. 8 top row). On the contrary, for the vaccine effect on the probabilities of needing an ICU when hospitalized and dying when in the ICU, severe epidemic and insufficient vaccine supply rate, the 42-day inter-dose period is more advantageous than 21 days at any plausible vaccine efficacy combination: if the first dose is more than about 50% efficacious, it is beneficial to delay the second dose by other three weeks at any efficacy of the second dose (Fig. 8 bottom row).

### Effects of vaccine efficacy waning

**Figure 9:**
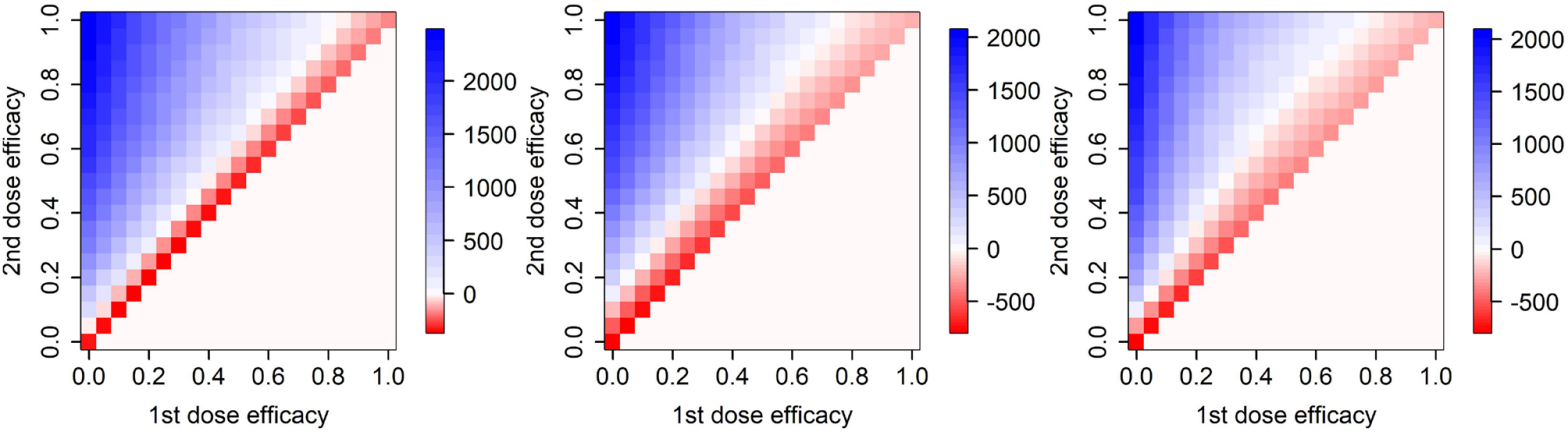
Efficacy of delaying the second vaccine dose by 3 weeks from 21 to 42 days, assuming waning vaccine efficacy against infection and symptoms appearance with $v_1^w=0.1$, $v_2^w=0.2$ (left), against infection and symptoms appearance with $v_1^w=0.2$, $v_2^w=0.3$ (middle), and with all considered modes of action together (right, $v_1^w=0.2$, $v_2^w=0.3$). Only model H is used here.

In addition, we test effects of waning of vaccine efficacy against infection and symptoms appearance. Thus, we assume that vaccine efficacy against infection and symptoms appearance may decline with time: step declines in efficacy by $v_1^w$ and $v_2^w$ are assumed to occur 28 days after the first dose and 90 days after the second dose, respectively. This means that the vaccine efficacy decline after the first dose is relevant only when the 42 days delay in administering the second dose is considered. As Fig. 9 demonstrates, waning considerations enlarge the (red) area in which it is preferrable to keep the second vaccine dose delay at 42 days. However, this enlargement is not quite dramatic even for relatively large and unrealistic vaccine efficacy declines. On the other hand, including also the vaccine action against hospital admission, need of ICU and death, kept constant over time, works against that enlarged 42-days-delay preference (Fig. 9).

## Discussion

The limited supply of COVID-19 vaccines around the world has called for a search for ways to distribute them to a larger number of people. One such strategy, widely used in Europe, was to increase the period between the first and second dose of vaccines with a two-dose scheme. This strategy, based on the idea that it is better to vaccinate more people with just one dose, even if full vaccine efficacy is not reached, rather than to put aside one dose for the re-vaccination for each administered one, is at a glance reasonable. As found by a study led by the University of Birmingham, an antibody response in adults 80+ years is 3.5 times larger in individuals that got the second dose of the Pfizer/BioNTech vaccine after 12 weeks compared to those who got it after 21 days^25^.

The argument that delaying the second vaccine dose is advantageous has received some support also via mathematical models^11,12^. Both these studies suggest that such a dose-sparing strategy would avert a significant proportion of infections that would otherwise occur if the originally recommended two-dose vaccination scheme is kept, provided that roughly 50% protection is achieved by the first dose. On the other hand, both studies are rather simple in many of their assumptions. The study of^12^ does not account for epidemic dynamics, spans a short time period and as the main comparative statistic considers the numbers of averted COVID-19 cases. The study of^11^, while based on a SEIR-type epidemic model, does not consider any dynamics in vaccine supply and is based on an artificial epidemic, assuming excessively high effective reproductive numbers ($R=1.8-2.1$). Moreover, neither of these studies considers various ways in which this vaccine may act.

Here we attempted to complement these studies and get further insight by: (i) considering a fully dynamic epidemic model calibrated for COVID-19 epidemic in Czechia, (ii) considering actual vaccination rollout scenarios adjusted for actual inter-dose periods and realistic supply rates, (iii) using the number of COVID-19-related deaths as the comparative statistic, (iv) considering various vaccine efficacies, epidemic severities, and ways in which vaccines may act. Our main conclusion is that vaccine effect on getting infected, mild epidemic and sufficient vaccine supply rate call for the original inter-delay scenario of 21 days regardless of the vaccine efficacy combination. On the contrary, for the vaccine effect on probability of needing an ICU and dying when on the ICU, severe epidemic and insufficient vaccine supply rate, the 42-day inter-dose period is more advantageous than 21 days at any plausible vaccine efficacy combination. In the spring of 2021, that was the case in many Asian and South American countries, as opposed to many European countries.

In^12^, the impact of vaccine shortages has a modal form: a moderate decrease in the vaccine supply rate implies more cases averted when the second dose is a bit postponed, but a larger reduction has a clear negative effect and should not lead to postponing the second dose administration. Also, that study shows that more severe epidemic should mean not prolonging the inter-dose period. Here we show just the opposite: lower vaccine availability and/or higher epidemic severity imply a stronger support for the inter-dose period of 42 days. We also show that postponing the second dose may be more effective even when the first dose efficacy is deeply under 50%, depending on its way of action, and that the efficacy of the second dose plays the role, too.

With quite some uncertainty regarding the way of vaccine action and the corresponding efficacy (or effectiveness) in the spring of 2021, the vaccine effectiveness values published in the literature have been more and more precise as new studies arose in response to intense vaccination campaigns in some countries^14–16,22,26,27^. Because of this uncertainty, we examined all possible combinations of the first and second dose efficacy/effectiveness. This general picture turns out useful also in the light of novel variants-of-concern that we have witnessed since, given variable efficacy/effectiveness of available vaccines to each of these variants^23,24,28^. Still, our study has some limitations. First, novel variants that have recently appeared are not only associated with diverse vaccine efficacies, but appeared in (or rather caused) different epidemic waves, have different values of $R_0$ and implied different hospitalization needs. Second, all this has varied from country to country. And third, post-vaccination immunity waning has recently become a new player in the game. We have coped with some of these issues in our study, but clealy a more detailed sensitivity analysis is desirable. Whereas^11^ used a simple generic model of epidemic dynamics, we considered a much detailed model calibrated to a real COVID-19 wave in Czechia. A model of intermediate complexity that would nonetheless consider all the essentials we cover here would likely be a promising next step in this respect.

In summary, we found that the vaccine effect at the beginning of infection progress (infection, symptom appearance), mild epidemic and sufficient vaccine supply rate call for the original inter-delay scenario of 21 days regardless of vaccine efficacy. On the contrary, for vaccine effect at the end of infection progress (severe symptoms, death), severe epidemic and low vaccine supply rate, 42-day inter-dose period is better, at any plausible vaccine efficacy. Moreover, we show that vaccine-induced immunity waning does not have an important effect on our results, since the six weeks delay is still not large enough to cause any significant decline^23,24^.

## Supplementary Information

Supplementary Information.

## Data Availability

Epidemiological data used in this study were provided by the Institute of Health Information and Statistics of the Czech Republic (https://onemocneni-aktualne.mzcr.cz/covid-19). Sociological data used in this study were provided by the PAQ Reseach agency (www.paqresearch.cz). Computer codes used to run models F, H and M, as well as datasets behind all published figures, are available from the authors upon request.

## References

[1] Giordano G (2021). Modeling vaccination rollouts, SARS-CoV-2 variants and the requirement for non-pharmaceutical interventions in Italy. Nat. Med..

[2] Moore S, Hill EM, Tildesley MJ, Dyson L, Keeling MJ (2021). Vaccination and non-pharmaceutical interventions for COVID-19: A mathematical modelling study. Lancet Infect. Dis..

[3] Amaku M, Covas DT, Coutinho FAB, Azevedo RS, Massad E (2021). Modelling the impact of delaying vaccination against SARS-CoV-2 assuming unlimited vaccines supply. Theor. Biol. Med. Model..

[4] Paltiel AD, Schwartz JL, Zheng A, Walensky RP (2021). Clinical outcomes of a COVID-19 vaccine: Implementation over efficacy. Health Aff..

[5] Fact sheet for healthcare providers administering vaccine (vaccination providers). www.fda.gov/media/146304/download. Accessed from 29 Mar 2022.

[6] COVID-19: Vaccination overview in the Czech Republic. https://onemocneni-aktualne.mzcr.cz/vakcinace-cr. Accessed from 29 Mar 2022.

[7] Use of COVID-19 vaccines in the United States: Interim clinical considerations. https://www.cdc.gov/vaccines/covid-19/info-by-product/clinical-considerations.html. Accessed from 29 Mar 2022.

[8] Specific situations regarding vaccination in the Czech Republic. https://covid.gov.cz/situace/informace-o-vakcine/specificke-situace-pri-ockovani. Accessed from 29 Mar 2022.

[9] Bubar KM (2021). Model-informed COVID-19 vaccine prioritization strategies by age and serostatus. Science.

[10] Moore S, Hill EM, Dyson L, Tildesley MJ, Keeling MJ (2021). Modelling optimal vaccination strategy for SARS-CoV-2 in the UK. PLoS Comput. Biol..

[11] Paltiel AD, Zheng A, Schwartz JL (2021). Speed versus efficacy: Quantifying potential tradeoffs in COVID-19 vaccine deployment. Ann. Intern. Med..

[12] Tuite AR, Fisman DN, Zhu L, Salomon JA (2021). Alternative dose allocation strategies to increase benefits from constrained COVID-19 vaccine supply. Ann. Intern. Med..

[13] Weiner, J., Blechová, E., Levinský, R. & Horká, R. Do kdy je možné naočkovat nejrizikovější skupiny? (2021). https://bit.ly/3a3wW5q. Accessed from 29 Mar 2022.

[14] Hall VJ (2021). COVID-19 vaccine coverage in health-care workers in England and effectiveness of BNT162b2 mRNA vaccine against infection (SIREN): A prospective, multicentre, cohort study. Lancet.

[15] Vasileiou E (2021). Effectiveness of first dose of COVID-19 vaccines against hospital admissions in Scotland: National prospective cohort study of 5.4 million people. SSRN Electron. J..

[16] Haas EJ (2021). Impact and effectiveness of mRNA BNT162b2 vaccine against SARS-CoV-2 infections and COVID-19 cases, hospitalisations, and deaths following a nationwide vaccination campaign in Israel: An observational study using national surveillance data. Lancet.

[17] Toni T, Welch D, Strelkowa N, Ipsen A, Stumpf MPH (2009). Approximate Bayesian Computation scheme for parameter inference and model selection in dynamical systems. J. R. Soc. Interface.

[18] Csilléry K, Blum MG, Gaggiotti OE, FrançSois O (2010). Approximate Bayesian computation (ABC) in practice. Trends Ecol. Evolut..

[19] Research, P. Life during pandemic (2020). www.zivotbehempandemie.cz. Long-term sociological panel survey. Accessed from 29 Mar 2022.

[20] Berec L (2021). Model-M: An agent-based epidemic model of a middle-sized municipality. medRxiv.

[21] Smid M (2021). SEIR filter: A stochastic model of epidemics. medRxiv.

[22] Dagan N (2021). BNT162b2 mRNA Covid-19 vaccine in a nationwide mass vaccination setting. New Engl. J. Med..

[23] Goldberg Y (2021). Waning immunity after the BNT162b2 vaccine in Israel. New Engl. J. Med..

[24] Tartof SY (2021). Effectiveness of mRNA BNT162b2 COVID-19 vaccine up to 6 months in a large integrated health system in the USA: A retrospective cohort study. Lancet.

[25] Delaying second Pfizer vaccines to 12 weeks significantly increases antibody responses in older people, finds study. https://www.birmingham.ac.uk/news/latest/2021/05/covid-pfizer-vaccination-interval-antibody-response.aspx. Accessed from 29 Mar 2022.

[26] Polack FP (2020). Safety and efficacy of the BNT162b2 mRNA Covid-19 vaccine. New Engl. J. Med..

[27] Skowronski DM, Serres GD (2021). Safety and efficacy of the BNT162b2 mRNA Covid-19 vaccine: Letter to the editor. New Engl. J. Med..

[28] Andrews N (2022). Covid-19 vaccine effectiveness against the Omicron (B.1.1.529) variant. New Engl. J. Med..

