# Supplementary material for: Importance of vaccine action and availability and epidemic severity for delaying the second vaccine dose
Source: Sci Rep. 2022 May 10;12:7638. doi: 10.1038/s41598-022-11250-4 (PMC9086670; doi:10.1038/s41598-022-11250-4)
Supplement: Supplementary file 1 — Supplementary Information. [file 41598_2022_11250_MOESM1_ESM.pdf]

Importance of vaccine action and availability and epidemic severity for delaying the second vaccine dose

Luděk Berec,[1,2*] René Levínský,[3] Jakub Weiner,[4] Martin Šmíd,[5] Roman Neruda,[6] Petra Vidnerová,[6] Gabriela Suchopárová [6]

[1]Centre for Mathematical Biology, Department of Mathematics, Faculty of Science, University of South Bohemia, Branišovská 1760, 37005 České Budějovice, Czech Republic
[2]The Czech Academy of Sciences, Biology Centre, Institute of Entomology, Department of Ecology, Branišovská 31, 37005 České Budějovice, Czech Republic
[3]CERGE-EI, Politických vězňů 7, 11121 Praha 1, Czech Republic
[4]Siesta Labs, Konopišťská 16, 10000 Praha 10, Czech Republic
[5]The Czech Academy of Sciences, Institute of Information Theory and Automation, Pod Vodárenskou věží 4, 18200 Praha 8, Czech Republic
[6]The Czech Academy of Sciences, Institute of Computer Science, Pod Vodárenskou věží 2, 18200 Praha 8, Czech Republic

*Corresponding author: lberec@prf.jcu.cz

Materials and Methods

All three models we use to address our research question are of the SEIR type, the generally accepted framework for modeling COVID-19, and all explicitly account for asymptomatic and presymptomatic infectious classes. That is, once infected, individuals are not infectious for a period. Once infectious, they either stay asymptomatic for the rest of infection (asymptomatic state) or are asymptomatic only for a short period (presymptomatic state) before symptoms eventually appear (symptomatic state). Symptomatic individuals may have only mild symptoms in which case they are (largely) isolated at home, or may have severe symptoms requiring hospitalization. Some hospitalized individuals eventually die. In addition, all three models are structured by age of individuals and type of inter-individual contacts. On the other hand, each model has a number of unique assumptions and characteristics. Here we describe Model H that focuses on dynamics in hospitals, while referring to publicly available descriptions of Model M [1] and Model F [2].

Description of Model H

Epidemic model Due to contacts with infectious individuals, susceptible individuals (class S) may become exposed (E), that is, infected but not yet infectious, with probability λ corresponding to the force of infection. The exposed individuals then become asymptomatic for the whole course of infection (A, with probability $1 - p_S$) or presymptomatic for just a short period of time before becoming symptomatic (P, with probability p_S). Average lengths of exposed, asymptomatic and presymptomatic periods are d_E, d_A and d_P days, respectively. The P individuals then become symptomatic (I), reducing their contacts with others by a factor r_C (imperfect social isolation). The A individuals eventually recover (R).

After an average period of d_I days, a proportion p_H of symptomatic individuals (those with relatively severe symptoms) are hospitalized (H). The remaining proportion $1 - p_H$ of symptomatic individuals (those that have only mild symptoms) remain isolated at home, staying in class I_R until recovery, with contacts again reduced by r_C, which lasts on average $d_A - d_P - d_I$ days. Here we assume that the average period from infection to recovery is the same for individuals with no or mild symptoms. And since the period from appearance of symptoms to recovery differs from that from symptoms occurrence to hospitalization, and the probability of hospitalization p_H is independent of these periods, we need to introduce an artificial inter-class I_R.

Hospitalized individuals follow two different pathways. After d_{HJ} days spent on a common hospital bed, a proportion p_J of such individuals require intensive care (J). Individuals that do not need intensive care go after those d_{HJ} days to another inter-class H_R where they eventually recover after next d_{HR} days. While on an ICU, individuals may die (D) with probability p_D (after d_{JD} days). If not dying, which happens with the complementary probability $1 - p_D$, they eventually recover, after spending further d_{JH} days on the ICU (inter-class J_H) and other d_{HR2} days on the common bed (H_{R2}). All model variables are summarized in Table 1.

Notation	Description
S	Susceptible individuals
E	Exposed individuals
A	Asymptomatic individuals for the whole course of infection
P	Presymptomatic individuals before becoming symptomatic
I	Symptomatic individuals
I_R	Symptomatic individuals that continue to stay in isolation
H	Hospitalized individuals initially on common bed
J	Hospitalized individuals on ICU
H_R	Hospitalized individuals that continue on common bed
D	Individuals that die when on ICU
J_H	Hospitalized individuals that continue on ICU
H_{R2}	Hospitalized individuals after leaving ICU
R	Recovered individuals

Table 1. List of variables used in Model H.

Finally, the SARS-CoV-2 virus is known to differently impact various age cohorts [3]. Therefore, we distinguish four age cohorts, 0-19 years (named children, coded 1), 20-64 years (adults, 2), 65-79 years (seniors, 3), and 80+ years (elderly, 4). These classes interact via the force of infection (see below). Once infected, individuals of each age cohort proceed independently of individuals of other age cohorts. Nonetheless, some model parameters used to decide on specific pathways through the model are age-specific. These are probabilities of becoming symptomatic p_S, requiring hospitalization p_H, needing ICU p_J, and dying p_D, but also all periods of staying in various hospital classes.

In discrete time, with one time step corresponding to one day, the above model description can be translated into the following system of equations:

$$
\begin{aligned}
S[t+1,a] &= S[t,a] - \lambda[t]\, S[t,a], \\
E[t+1,a] &= E[t,a] + \lambda[t]\, S[t,a] - \sigma E[t,a], \\
A[t+1,a] &= A[t,a] + (1-p_S[a])\, \sigma E[t,a] - \gamma_A A[t,a], \\
P[t+1,a] &= P[t,a] + p_S[a]\, \sigma E[t,a] - \xi P[t,a], \\
I[t+1,a] &= I[t,a] + \xi P[t,a] - \alpha_I I[t,a], \\
I_R[t+1,a] &= I_R[t,a] + (1-p_H[a])\, \alpha_I I[t,a] - \gamma_{IR} I_R[t,a], \\
H[t+1,a] &= H[t,a] + p_H[a]\, \alpha_I I[t,a] - \alpha_H[a]\, H[t,a], \\
H_R[t+1,a] &= H_R[t,a] + (1-p_J[a])\, \alpha_H[a]\, H[t,a] - \gamma_{HR}[a]\, H_R[t,a], \\
J[t+1,a] &= J[t,a] + p_J[a]\, \alpha_H[a]\, H[t,a] - \alpha_J[a]\, J[t,a], \\
D[t+1,a] &= D[t,a] + p_D[a]\, \alpha_J[a]\, J[t,a], \\
J_H[t+1,a] &= J_H[t,a] + (1-p_D[a])\, \alpha_J[a]\, J[t,a] - \alpha_{JH}[a]\, J_H[t,a], \\
H_{R2}[t+1,a] &= H_{R2}[t,a] + \alpha_{JH}[a]\, J_H[t,a] - \gamma_{HR2}[a]\, H_{R2}[t,a], \\
R[t+1,a] &= R[t,a] + \gamma_{HR2}[a]\, H_{R2}[t,a] + \gamma_{HR}[a]\, H_R[t,a] + \gamma_{IR} I_R[t,a] + \gamma_A A[t,a],
\end{aligned}
\tag{1}
$$

where all variables are functions of time t and age cohort $a = 1,2,3,4$. The model parameters σ, ξ, $\alpha_{I/H/J/JH}$, and $\gamma_{A/IR/HR/HR2}$ represent probabilities with which individuals leave respective model classes. These probabilities are related to the average periods an individual spends in each such class (Table 2).

Notation	Description	Relationship to delays
σ	Probability of leaving E class	$1-\exp(-1/d_E)$
ξ	Probability of leaving P class	$1-\exp(-1/d_P)$
α_I	Probability of leaving I class for H or I_R classes	$1-\exp(-1/d_{IH})$
α_H	Probability of leaving H class for J or H_R classes	$1-\exp(-1/d_{HJ})$
α_J	Probability of leaving J class for D or J_H classes	$1-\exp(-1/d_{JD})$
α_{JH}	Probability of leaving class J_H for class H_{R2}	$1-\exp(-1/d_{JH})$
γ_A	Probability of leaving class A for recovery	$1-\exp(-1/d_A)$
γ_{IR}	Probability of leaving class I_R for recovery	$1-\exp(-1/d_{IR})$
γ_{HR}	Probability of leaving class H_R for recovery	$1-\exp(-1/d_{HR})$
γ_{HR2}	Probability of leaving class H_{R2} for recovery	$1-\exp(-1/d_{HR2})$

Table 2. Probabilities of leaving particular model classes calculated from average periods spent in those classes.

Force of infection The force of infection λ in the model (1) sums contributions from all infectious classes (A, P, I, I_R) across all age cohorts we consider:

$$\lambda[t] = \sum_{k=1}^{4} w_\beta[t]\,\beta[k] \sum_{l=1}^{4} C[t,l,k]\,\frac{r_\beta A[t,l] + P[t,l] + r_C I[t,l] + r_C I_R[t,l]}{N[t,l]}. \quad (2)$$

Here, $\beta[k]$ is the age-specific probability that a susceptible individual from age cohort k is infected by a (sufficiently close and lengthy) with an infectious individual of any age cohort, $C[t,l,k]$ is the contact rate (the mean number of individuals of age l that an individual of age k has an effective contact with during day t), r_β is a factor reducing the infection transmission probability for an asymptomatic individual relative to a (pre)symptomatic, r_C is a factor reducing the contact rate of a symptomatic individual relative to an a/presymptomatic one (having symptoms should force an individual to reduce contacts with others but in reality isolation is imperfect), and $N[t,l]$ is the number of 'active' age l individuals at time t, that is, those except dead, in hospitals and symptomatic individuals that are isolated. All parameters and their values we run the model with are given in Table 3.

In addition, $w_\beta[t]$ is the time-varying (but age-independent) factor reducing (in case of personal protective measures such as masks) or enhancing (in case of more contagious viral variants) the probability of infection transmission upon contact β. We may thus view it as a product of reducing and enhancing forces. We set the reducing force to 0.3 until February 28, 2020, and decrease it further to 0.23 from March 1, 2021, the day from which FN95 respirators were ordered to wear instead of common medical masks. We calculate these numbers as

$$r_P^2\,p_P^2 + 2\,r_P\,(1-r_P)\,p_P + (1-r_P)^2, \quad (3)$$

where $r_P = 0.6$ represents 60% compliance of using personal protection measures and p_P is the proportional reduction in transmission due to personal protection measures. We set p_P to 0.25 until February 28, 2020, and to 0.125 from March 1, 2021. Moreover, we increase the enhancing force from 1 to 1.5 since January 1, 2021, to March 1, 2021, to account for invasion and eventual domination of the B.1.1.7 variant of SARS-CoV-2 virus in the Czech Republic.

Finally, construction of the time-varying contact matrix C starts with [4], a study in which such a matrix was published for 152 countries, including Czechia, for the pre-pandemic times. They expressed it as a sum of four specific contact matrices describing daily numbers of contacts at home (C_H), school (C_S), work (C_W), and of other types of contact (C_O). We transform the Czech matrices of [4], structured by 5-year age classes, to fit our three age cohorts. This is because data in [4] end with the 75-80 years age class. To supply values for our age cohort 4 (adults 80+ years), we replicate data for our age cohort 3 (adults 65-79 years) to our age cohort 4, too, to get:

$$C_H = \begin{bmatrix} 1.52 & 0.67 & 0.036 & 0.036 \\ 2.84 & 2.05 & 0.20 & 0.20 \\ 0.93 & 0.58 & 0.75 & 0.75 \\ 0.93 & 0.58 & 0.75 & 0.75 \end{bmatrix}, \quad C_S = \begin{bmatrix} 4.77 & 0.20 & 0.0014 & 0.0014 \\ 1.81 & 0.33 & 0.0075 & 0.0075 \\ 0.022 & 0.019 & 0.022 & 0.022 \\ 0.022 & 0.019 & 0.022 & 0.022 \end{bmatrix}, \quad (4)$$

$$C_W = \begin{bmatrix} 0.085 & 0.19 & 1.4\times10^{-5} & 1.4\times10^{-5} \\ 0.42 & 5.28 & 9.4\times10^{-5} & 9.4\times10^{-5} \\ 1.75\times10^{-5} & 0.00012 & 4\times10^{-5} & 4\times10^{-5} \\ 1.75\times10^{-5} & 0.00012 & 4\times10^{-5} & 4\times10^{-5} \end{bmatrix}, \quad C_O = \begin{bmatrix} 1.61 & 0.78 & 0.24 & 0.24 \\ 1.10 & 3.94 & 1.01 & 1.01 \\ 0.15 & 0.89 & 0.93 & 0.93 \\ 0.15 & 0.89 & 0.93 & 0.93 \end{bmatrix}. \quad (5)$$

The time-varying contact matrix C is then calculate as a weighted sum of these four specific matrices,

$$C[t] = s_H[t]\,C_H + s_W[t]\,C_W + s_S[t]\,C_S + s_O[t]\,C_0,$$

where the weights $s_X, X = H, W, S, O$ are time-varying (changing on a weekly basis) proportional reduction factors due to adopted interventions and their compliance, estimated from panel surveys conducted by the PAQ Reseach agency (www.zivotbehempandemie.cz; Fig.1).

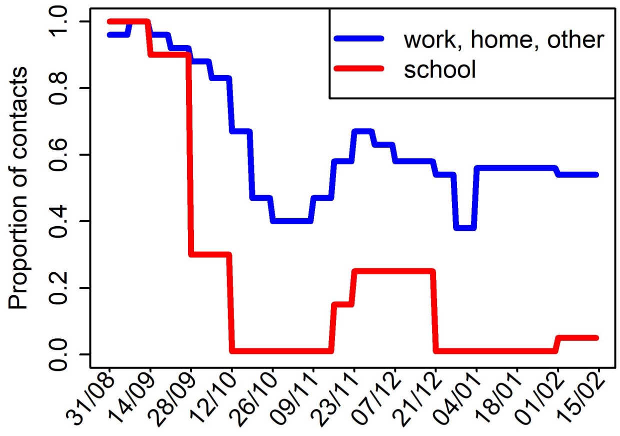

Figure 1. Proportional reductions in different types of contact evolving in time on a weekly basis. Based on panel surveys conducted by the PAQ Reseach agency (www.zivotbehempandemie.cz).

Initial conditions Our simulations start on August 31, 2020, the day around which the second COVID-19 wave in Czechia presumably began and also corresponds to Monday (contact data described above are provided for calendar weeks). Whereas initial values of state variables related to hospitals are inferred from data, initial values for the remaining (hidden) state variables (E, A, P, I, I_R) are regarded as parameters to which the model is calibrated; see below for the calibration procedure.

Vaccination Only susceptible individuals are vaccinated. When vaccinated, individuals pass through three sequential vaccination classes. Just vaccinated individuals enter the class with no vaccine effect in which they stay on average for two weeks. If not infected during this time, they pass to the second class for until one week after the second dose; the vaccine efficacy is v_e^1 when in this class. Eventually, if still not infected, vaccinated individuals pass to the third class in which vaccine efficacy is v_e^2. Vaccinated individuals can be infected when in any of these three classes. Once this happens, they stay in the current class and go through the sequence of states analogous to that for non-vaccinated infected individuals.

With these three classes of vaccinated individuals, the force of infection changes to

$$\begin{aligned}\lambda[t] = \textstyle\sum_{k=1}^{4} w_\beta[t]\,\beta[k]\,\sum_{l=1}^{4} C[t,l,k]\,\big(r_\beta\,(A[t,l]+A^v[t,l]) + (P[t,l]+P^v[t,l]) + \\ + r_C\,(I[t,l]+I^v[t,l]) + r_C\,(I_R[t,l]+I_R^v[t,l])\big)\,/N[t,l],\end{aligned} \tag{6}$$

where we assume that the infected vaccinated individuals are infectious to the same extent as the infected non-vaccinated individuals, and where the terms with the upper index v are numbers of the respective individuals over all three vaccination classes.

The ways vaccine is in this study assumed to acts in four ways (and some of their combination). These ways and the manner in which they are modeled are as follows:

(1) Reduction of the probability of getting infected upon contact with an infectious individual: the force of infection $\lambda[t]$ is reduced by the factor $1 - v_e^i$;

(2) Reduction of the probability of becoming symptomatic when infected: the probability p_S is reduced by the factor $1 - v_e^i$;

(3) Reduction of the probability of getting hospitalized when symptomatic: the probability p_H is reduced by the factor $1 - v_e^i$;

(4) Reduction of the probability of needing ICU when getting hospitalized: the probability p_J is reduced by the factor $1 - v_e^i$;

(5) Reduction of the probability of dying when on ICU: the probability p_D is reduced by the factor $1 - v_e^i$.

Model calibration Many model parameters can be taken from the literature or estimated from data provided by the Institute of Health Information and Statistics of the Czech Republic (UZIS); `www.uzis.cz`. However, values of some model parameters will always remain uncertain, of which the transmission probabilities $\beta[i]$, $i = 1,2,3,4$, are commonly of this kind. These and some other model parameters, listed in Table 3, are estimated by fitting the simulated cumulative numbers of deaths and the actual numbers of hospitalized individuals in age classes 2, 3 and 4 to the corresponding age-specific actual time series collected in the Czech Republic.

Many optimization and filtering methods have been developed to meaningfully perform model calibration [5]. Here we adopted the Approximate Bayesian Computation (ABC) technique, used to estimate parameters of complex models in genomics and other biological disciplines, including epidemiology [6, 7, 8, 9, 10]. The major advantage of this method is that it naturally works with all sources of uncertainty acknowledged in the model. At the same time, the ABC does not rely on likelihood calculation and in case of sufficient computation power can be used with models of virtually any complexity.

The variant of ABC with rejection sampling that we used consisted of three steps. First, we performed $K = 200,000$ model simulations, collecting the age-specific cumulative numbers of deaths (D) and actual numbers of hospitalized individuals ($H + H_R + J + J_H + H_{R2}$), drawing values of the uncertain model parameters from prior distributions based on literature and available data on the Czech Republic epidemic; selected prior distributions for the parameters to be estimated are given in Table 3. Second, we calculated summary statistics on the simulated and corresponding observed time series, using their Euclidean distance D. Third, we selected model simulations that satisfied $D < \varepsilon$, where ε was chosen to pass 0.025% (50) of the simulations into the selected set. Since the used summary statistics are informative, the distribution of parameters corresponding to the selected simulations is known to converge from outside to the Bayesian posterior distribution of parameter values with N going to infinity and ε going to 0, and is referred to as the approximate posterior [7]. The choice of N and ε in the ABC is driven by compromise between computation power and smoothness and accuracy of the approximate posterior.

The set of selected parametric sets thus allows us to evaluate remaining parameter uncertainty, given the available data and adopted summary statistics [6, 7, 8]. This is crucial to realize, since although different parameter sets may similarly fit the available data (have similar summary statistics), and often provide similar short-term predictions, they may demonstrate significant differences in longer term and in interplay with intervention policies. To apply the ABC technique, we use the `abc` package in R [11], modified to work with non-normalized summary statistics.

Parameter	Meaning	Value	Reference
$\beta_1,\beta_2,\beta_3,\beta_4$	Age-specific transmission probabilities	uniform on $[0.01, 0.99]$	calibration
r_β	Factor reducing infection transmission from asymptomatic individuals	0.5	[3]
r_C	proportional contact reduction in isolated individuals	uniform on $[0.01, 0.4]$	calibration
p_S	proportion symptomatic	uniform on $[0.65, 0.84]$	calibration, halved for the 0-19 years age cohort
p_H	proportion hospitalized among symptomatic	$(0.0052, 0.056, 0.270.43)$	UZIS
p_J	proportion on ICU among hospitalized	uniform on $(0-0.02, 0-0.3, 0.1-0.4, 0.1-0.7)$	UZIS $(0, 0.16, 0.24, 0.19)$, calibration
p_D	proportion dying on ICU	uniform on $(0-0.02, 0.1-0.5, 0.3-0.9, 0.5-0.9)$	UZIS $(0, 0.24, 0.51, 0.74)$, calibration
d_E	latent period	uniform on 3-7 days	calibration
d_A	time to recovery when asymptomatic	uniform on 8-14 days	calibration
d_P	presymptomatic period	uniform on 2-6 days	calibration
d_{IH}	period from symptoms appearance	$(5.71, 7.41, 5.87, 5.87)$ days	UZIS
d_{IR}	recovery time when isolated	$d_A - d_P - d_{IH}$	–
d_{HJ}	period from hospital to ICU admission	$(1.88, 3.01, 3.85, 4.50)$	UZIS
d_{JD}	time on ICU till death	$(10.0, 9.41, 7.89, 4.43)$	UZIS
d_{JH}	extra time on ICU when not dying	$(0, 0, 0.71, 2.22)$	UZIS
d_{HR2}	time from leaving ICU to recovery	$(3.08, 4.51, 4.95, 6.12)$	UZIS

Table 3. Parameters of Model H. UZIS = The Institute of Health Information and Statistics of the Czech Republic (`www.uzis.cz`); mean values are provided over the period from August 31, 2020, until February 14, 2021. Distributions of all the indicated delays were checked and all are exponential. Times are given in days. The indicated distributions are prior distributions for the calibration procedure, and are based on the literature.

References

1. Berec, L. *et al.* Model-M: An agent-based epidemic model of a middle-sized municipality. *medRxiv;* 10.1101/2021.05.13.21257139 (2021).
2. Smid, M. *et al.* SEIR filter: a stochastic model of epidemics. *medRxiv;* 10.1101/2021.02.16.21251834 (2021).
3. Davies, N. G. *et al.* Age-dependent effects in the transmission and control of COVID-19 epidemics. *Nature Medicine* **26**, 1205–1211 (2020).
4. Prem, K., Cook, A. R. & Jit, M. Projecting social contact matrices in 152 countries using contact surveys and demographic data. *PLoS Computational Biology* **13**, e1005697 (2017).
5. Yang, W., Karspeck, A. & Shaman, J. Comparison of filtering methods for the modeling and retrospective forecasting of influenza epidemics. *PLoS Computational Biology* **10**, e1003583; 10.1371/journal.pcbi.1003583 (2014).
6. Toni, T., Welch, D., Strelkowa, N., Ipsen, A. & Stumpf, M. P. H. Approximate Bayesian Computation scheme for parameter inference and model selection in dynamical systems. *Journal of The Royal Society Interface* **6**, 187–202 (2009).
7. Beaumont, M. A. Approximate Bayesian Computation in evolution and ecology. *Annual Review of Ecology, Evolution, and Systematics* **41**, 379–406 (2010).
8. Csilléry, K., Blum, M. G., Gaggiotti, O. E. & François, O. Approximate Bayesian Computation (ABC) in practice. *Trends in Ecology and Evolution* **25**, 410–418 (2010).

9. Blum, M. G. & Tran, V. C. HIV with contact tracing: a case study in approximate Bayesian computation. *Bioinformatics* **11**, 644–660 (2010).

10. Luciani, F., Sisson, S. A., Jiang, H., Francis, A. R. & Tanaka, M. M. The epidemiological fitness cost of drug resistance in *Mycobacterium tuberculosis*. *PNAS* **106**, 14711–14715 (2009).

11. Csilléry, K., Lemaire, L., François, O. & Blum, M. G. abc: tools for Approximate Bayesian Computation (ABC) (2015). URL `cran.r-project.org/web/packages/abc/index.html`. Accessed: 2022-03-29.
